# Left ventricular function changes and echocardiographic predictors in adult survivors of fulminant myocarditis treated with the Chinese protocol

**DOI:** 10.1038/s41598-023-33285-x

**Published:** 2023-04-18

**Authors:** Luying Jiang, Kaiyue Zhang, Chunran Zhang, Yujian Liu, Jiangang Jiang, Daowen Wang, Houjuan Zuo, Hong Wang

**Affiliations:** 1 Division of Cardiology, Department of Internal Medicine, Tongji Hospital, Tongji Medical College, Huazhong University of Science and Technology, Wuhan, 430030 People’s Republic of China; 2 Hubei Key Laboratory of Genetics and Molecular Mechanisms of Cardiologic Disorders, Wuhan, 430030 Hubei Province People’s Republic of China; 3 The 3rd Department of Cardiology, The First Affiliated Hospital of The Medical College, Shihezi University, Shihezi, Xinjiang 832008 People’s Republic of China

**Keywords:** Cardiology, Diseases

## Abstract

Disagreement exists regarding the long-term prognosis and recovery of left ventricular (LV) function in patients with fulminant myocarditis (FM). This study reported the outcome and LV ejection fraction (EF) changes in FM treated with Chinese protocol, and assessed whether global longitudinal strain (GLS) by two-dimensional speckle tracking echocardiography (2-D STE) could provide additional information. This retrospective study included 46 FM adult patients who applied timely circulatory support and immunomodulatory therapy with adequate doses of both glucocorticoids and immunoglobulins as core approaches and survived after acute phase. They all presented with acute onset of cardiac symptoms < 2 weeks. LV end-diastolic dimensions, LVEF and GLS at discharge and 2-year were obtained and compared. We then performed linear regression and ROC analysis to determine independent factors to predict normalization of GLS at 2-year. At 2 years, the survival was 100% in our cohort. And the GLS improved modestly (15.40 ± 3.89% vs 17.24 ± 2.89%, *P* = 0.002). At two years, a proportion of patients whose LV function remained abnormal, being 22% evaluated by EF (< 55%) and higher to 37% by GLS (< 17%). Moreover, GLS at discharge but not at presentation correlated with GLS at 2-year (r = 0.402, *P* = 0.007). The FM adult treated with Chinese protocol have good survival and modest improvement of LV function during 2-year.

## Introduction

Myocarditis is an inflammatory disease of heart with a wide spectrum of clinical presentations and outcomes. A proportion of them can present with sudden onset, prompt hemodynamic compromise, rapidly progressing and severely impaired cardiac function, and require inotropic agents or mechanical circulatory supports (MCS)^1,2^. Clinically they are labelled as fulminant myocarditis (FM), but have different pathogenesis with lymphocytic myocarditis as the most frequent form^3^. Compared with non-fulminant myocarditis (NFM), FM patients have a remarkably higher risk of death in the near term, varying from 16.7% to about 50% in different reports^4–7^. Our center applied the Chinese protocol, Life Support Based Comprehensive Treatment (LSBCTR), in FM and lowered in-hospital mortalities to < 4%^8,9^, although it was still higher than that of NFM^4^. The Life Support Based Comprehensive Treatment focuses on MCS, immunomodulatory agents (hormones and immunoglobulins), and particularly emphasizes "early recognition, early diagnosis, early prognosis, and early treatment".

In comparison with the poor short-term outcomes, the long-term prognosis in FM remains controversial. An early publication in 2000 by McCarthy et al. paradoxically reported better long-term outcomes in FM, and proposed that FM will have a benign prognosis once they survive in the index hospitalization. In contrast, recent evidences from two contemporary studies by Ammirati et al. refuted this early conclusion. In this and other studies, the rate of death and heart transplantation for in-hospital period, mid-term (60 days) and long-term (7 or 9 years) were all consistently higher in FM than in NFM^3,4,10^. Moreover, the course of FM after the acute episode and its transition to chronic dilated cardiomyopathy remains poorly understood. Studies evaluating left ventricular (LV) remodelling and functional changes in FM are also lacking.

Our multi-centre study applied the Chinese protocol in treating FM, and as a result dramatic reduction of in-hospital mortality^8^ and steep improvement of cardiac function at discharge were observed^11^. Recently, Hang et al. have also shown that no death was observed at one-year follow-up, proving favourable survival in Chinese FM cohort^12^. In this 2-year follow-up study, other than survival, we evaluated the LV structural and functional changes in a larger cohort of patients with viral FM who survived the acute episode. The global longitudinal strain (GLS) by two-dimensional speckle tracking (2-D STE) which is a more accurate and sensitive indicator than EF in evaluating cardiac function^13^. And it’s more informative in FM^11,14^ to determine if it can provide additional information on LV functional changes in FM. Furthermore, potential early predictors of long-term LV functional recovery were explored.

## Results

### Clinical characteristics

The main clinical characteristics of FM patients were presented in Supplemental Table 1. The mean age was 33 ± 13 years with 23 males and 23 females. Peak troponin-T value was 39,649.75 (14,830.75, 50,000.00) pg/ml, and Troponin-T value at discharge was 153.15(73.98, 374.18) pg/ml. IABP was applied in 46 (100%) and ECMO in 9 (20%) patients. Endomyocardial biopsy (EMB) was performed in 15 (33%) patients. And all patients underwent CMR before discharge to confirm myocardial inflammatory damages. Moreover, there were no death or heart transplantation at 2-year follow-up for all 46 patients in the study.

### Comparison of echocardiographic features at discharge with that at 2-year

**Figure 1:**
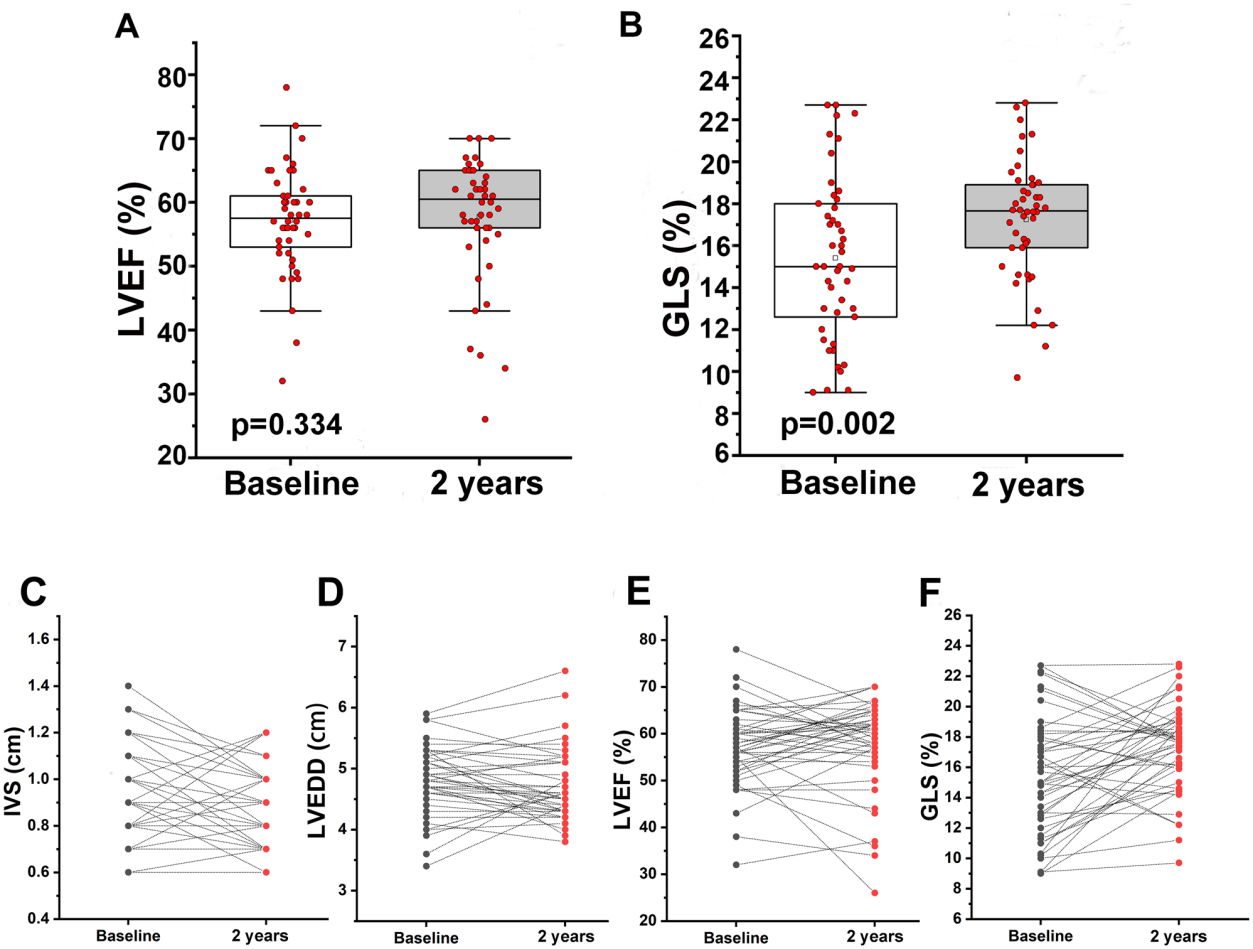
Comparison of LVEF and GLS at discharge with that at 2-year follow-up in FM. (**A** and **B**) There was a significant improvement for GLS (15.40 ± 3.89 vs 17.24 ± 2.89, p = 0.002) but not for LVEF (57.20 ± 8.29 vs 57.74 ± 9.82, p = 0.334). Individual changes of IVS, LVEDD, LVEF and GLS at discharge and at 2-year were also shown (**C**–**F**). http://m.qpic.cn/psc?/V53wpKJ24F3mOO015TIE1dJF3728Qwlj/ruAMsa53pVQWN7FLK88i5oJoe1CbnGP7nopAtC78vvMNdP5HrsP7pgz*HPB8qe4QGVWxGDy1Y838JPaEnpWfjqvykTmC9ptGfPrfQBxtjeo!/mnull&bo=gAdABoAHQAYBByA!&rf=photolist&t=5.

The standard echocardiogram and strain analysis was performed in FM survivors at discharge and at 2-year follow-up and the results were shown in Table 1. There was no significant difference between at discharge and at 2-year for both the LV end-diastolic dimensions (LVEDD) (4.78 ± 0.55 cm vs. 4.71 ± 0.56 cm, *P* = 0.281) (Fig. 1D) and LVEF (57.20 ± 8.29% vs. 57.74 ± 9.82%, P = 0.334) (Fig. 1A,E). However, the interventricular septal thickness was thinner at 2-year, compared with that at discharge (1.12 ± 0.23 cm vs. 1.25 ± 0.21 cm,* P* = 0.005) (Fig. 1C).

**Table 1:** Comparison of standard echocardiographic and strain analysis parameters between at discharge and at 2-year follow-up.

Parameters	At discharge	At 2-year follow-up	P
IVS diastolic (cm)	1.07 ± 0.76	0.86 ± 0.17	0.086
IVS systolic (cm)	1.25 ± 0.21	1.12 ± 0.23	0.005
LVEDD (cm)	4.78 ± 0.55	4.71 ± 0.56	0.281
LVESD (cm)	3.47 ± 0.61	3.38 ± 0.69	0.182
End-diastolic volume (mL)	125.41 ± 40.17	112.64 ± 34.15	0.085
End-systolic volume (mL)	58.25 ± 29.69	50.40 ± 25.00	0.067
LA diameter (cm)	3.27 ± 0.57	3.25 ± 0.45	0.733
EF (%)	57.20 ± 8.29	57.74 ± 9.82	0.334
E/A	1.58 ± 0.59	1.48 ± 0.61	0.394
E′	8.74 ± 2.38	8.89 ± 2.73	0.692
E/E′	10.07 ± 2.71	9.19 ± 2.36	0.093
GLS PSLS (%)	15.40 ± 3.89	17.24 ± 2.89	0.002

*IVS* interventricular septum, *LVEDD* end-diastolic dimensions, *LVESD* end-systolic dimensions, *LA* left atrium, *EF* ejection fraction, *E* peak early diastolic mitral flow velocity, *A* peak late diastolic mitral flow velocity, *E′* Spectral pulsed-wave Doppler–derived early diastolic velocity from the septal mitral annulus, *PSLS* peak systolic longitudinal strain, *Global PSLS* including apical, mid and basal PSLS%.

In comparison with LVEF, the GLS at 2-year was significantly higher than that at discharge (17.24 ± 2.89% vs. 15.40 ± 3.89%, P = 0.002) (Fig. 1B,F). The improvement was greater in the anteo-septal (17.40 ± 3.97% vs. 14.50 ± 5.95%, *P* = 0.029) and the posterior wall (15.85 ± 5.77% vs. 12.19 ± 9.38%, *P* = 0.014). Representative bull’s-eye displays of GLSs at discharge and at 2 years in 2 patients were shown in Supplemental Fig. 1.

### LVEF, LV end-diastolic dimension and GLS in a proportion of patients with FM remained abnormal at 2-year follow-up

**Figure 2:**
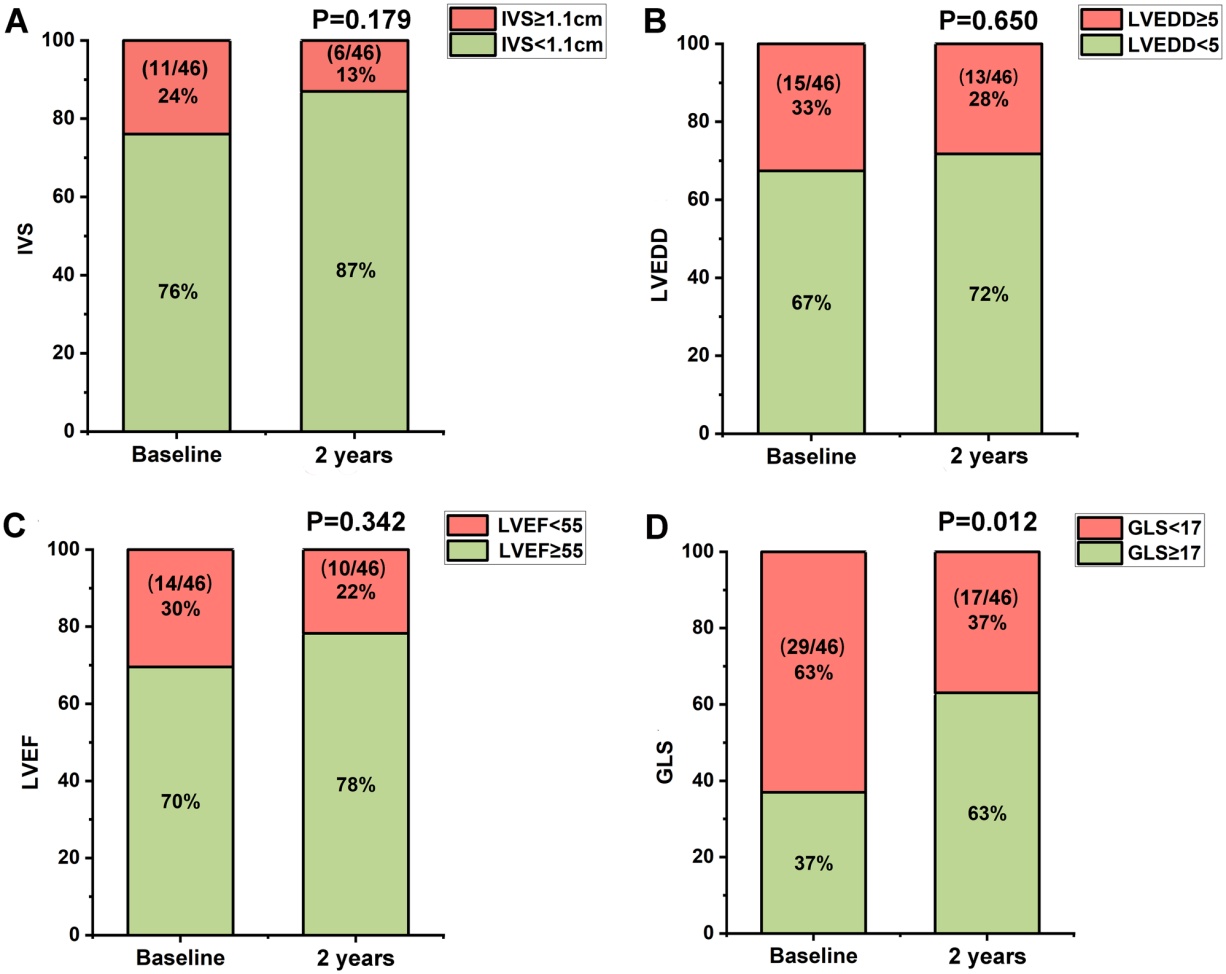
Changes in LV structural and functional parameters during 2-year follow-up in patients with FM. The proportion of FM patients with IVS < 1.1 cm (**A**) and LVEDD < 5 cm (**B**) at 2-year follow-up was higher than discharge. The proportion of patients with LVEF < 55% (**C**) and GLS < 17% (**D**) at 2-year follow-up was lower than discharge (Pearson test was used for comparison). http://m.qpic.cn/psc?/V53wpKJ24F3mOO015TIE1dJF3728Qwlj/ruAMsa53pVQWN7FLK88i5oJoe1CbnGP7nopAtC78vvPF7JLlGWMJHEjgyx.eGw3bT2kPtwNmkHV6l18AWl4XMK47GuPOF3JaC6qON*k4Z5k!/mnull&bo=gAdABoAHQAYBByA!&rf=photolist&t=5.

Although the LV function as represented by GLS in our FM cohort had modest improvement at 2-year after discharge, a proportion of them whose structural and functional indices remained abnormal at 2-year. At 2-year follow-up, there were 6 patients (13%) whose interventricular septum (IVS) was still ≥ 1.1 cm (Fig. 2A), and 13 patients (28%) whose LVEDD was ≥ 5.0 cm (Fig. 2B). Moreover, 10 of 46 (22%) patients whose LVEF remained < 55% (Fig. 2C) and 17 of 46 (37%) whose GLS remained < 17% (Fig. 2D). When GLS was considered, more patients had poor LV functional recovery at 2-year. Patients with poor recovery of LV function (PPR-LF) < 40% of LVEF are considered as PPR-LF, among whom adverse cardiovascular events usually occur. We found that 4 of 46 (9%) patients with LVEF < 40% at 2-year follow-up, two of whom were with LVEF < 40% at discharge.

Moreover, there were 2 subjects who had fully recovery of LVEF at discharge had dramatic decline of EF (a decrease > 10% and to < 50%) during their follow-up period. One of them had a decrease of LVEF from 57 to 43% and another one had LVEF reduction from 55 to 26%.

### GLS at discharge could predict normalization of GLS (≥ 17%) at 2-year

**Figure 3:**
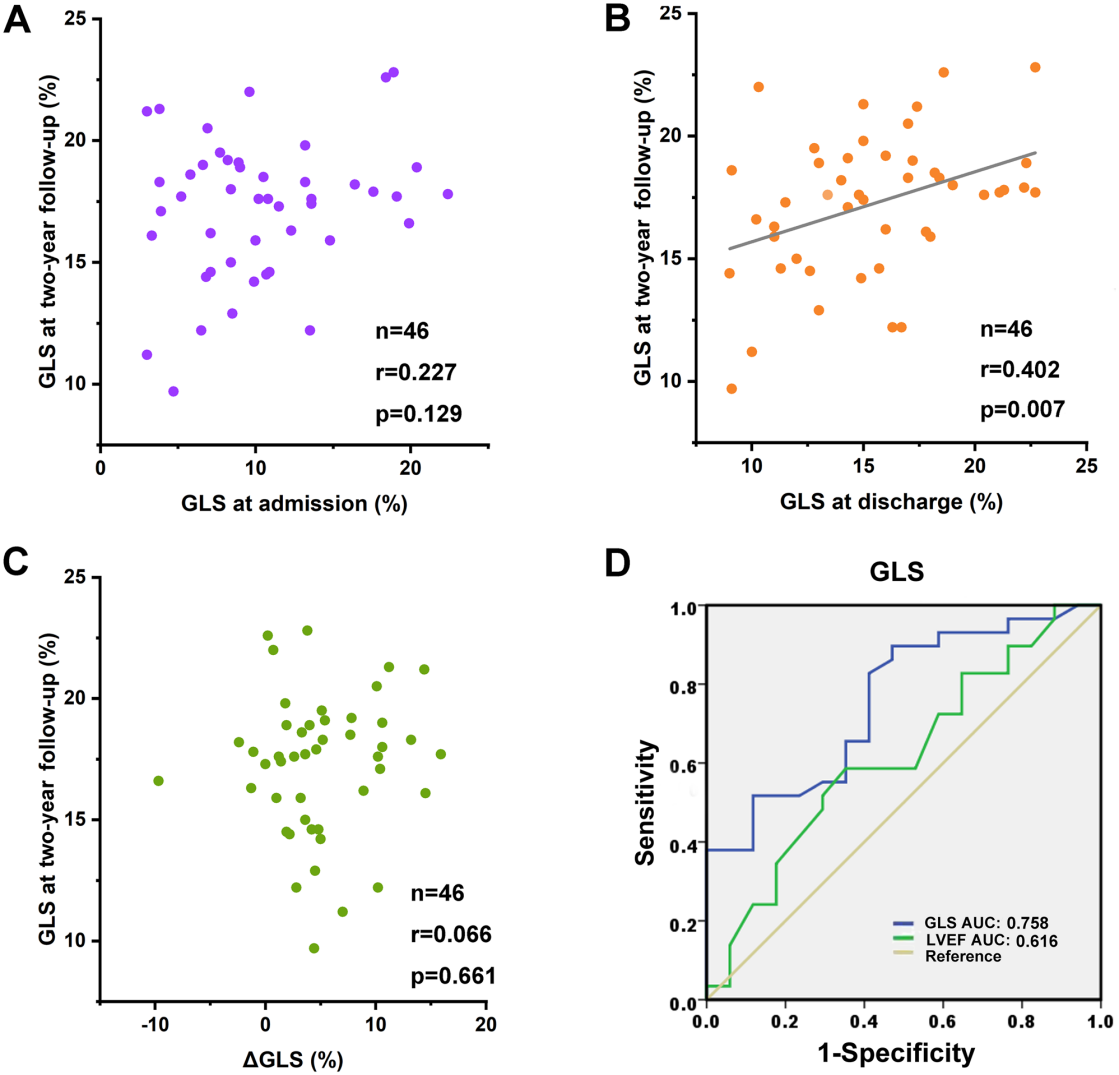
Dot plots of GLS at discharge, at admission and delta GLS (the difference of GLS between admission and discharge) in relation to GLS at 2-year follow-up. There was a significant correlation for GLS at discharge (**B**), but not GLS at admission (**A**) and delta GLS with GLS at 2-year (**C**). (**D**) Receiver operating characteristic curve analysis for the value of GLS at discharge to predict the GLS normalization at two years. GLS < 13.2% could predict the GLS normalization of (> 17%) with a sensitivity 82.2% and specificity of 58.8% (AUC: 0.758). AUC = area under curve. http://m.qpic.cn/psc?/V53wpKJ24F3mOO015TIE1dJF3728Qwlj/ruAMsa53pVQWN7FLK88i5oJoe1CbnGP7nopAtC78vvPOrRDrN2WJ5h8PF.TMtqLlrAytUJScEzyKAil910lHTF*JKfCKRsKRWzfEMe5k*Jo!/mnull&bo=gAdABoAHQAYBByA!&rf=photolist&t=5.

We further tested what indices could predict normalization of GLS by regression analysis. GLS ≥ 17% was used as normal reference according to previous data^15,16^. Univariate analysis was performed at first and results were shown in Table 2. We observed that LVEDD at discharge (r = − 0.337, P = 0.022), LVEF at discharge (r = 0.34, P = 0.021) and GLS at discharge (r = 0.379, P = 0.009) were significantly associated with the GLS normalization at 2-year. After correcting for BMI, systolic blood pressure, diastolic blood pressure, and left atrial diameter, only GLS at discharge (r = 0.402, *P* = 0.007) was found to be correlated with the GLS normalization at 2-year (Fig. 3B). Neither GLS at admission nor delta GLS (difference of GLS between admission and discharge) was correlated with the GLS normalization at 2-year by multivariate analysis (Fig. 3A,C).

**Table 2:** Linear regression analysis for GLS of 2-year follow-up in FM.

	Univariate		Multivariate	
	r	P-value	r	P-value
Age (year)	− 0.198	0.188		
Peak troponin-T (pg/mL)	− 0.08	0.596		
Troponin T (pg/mL)	− 0.209	0.164		
NT-proBNP (pg/mL)	0.088	0.561		
Glucocorticoid (mg)	− 0.227	0.129		
Days of glucocorticoid use (day)	− 0.087	0.566		
R-globulin (g)	− 0.159	0.292		
Days of r-globulin use (day)	− 0.128	0.398		
IABP-n (%)	− 0.048	0.75		
Days of IABP use (day)	− 0.135	0.371		
IABP + ECMO-n (%)	− 0.013	0.933		
IVS D (cm)	0.014	0.928		
LVEDD (cm)	− 0.337	0.022	− 0.187	0.315
LA D (cm)	− 0.159	0.292		
LV ejection fraction (%)	0.34	0.021	0.115	0.472
E-wave deceleration time (cm/s)	0.016	0.916		
E/A	− 0.103	0.494		
E/E′	− 0.111	0.463		
GLS at discharge (%)	0.379	0.009	0.402	0.007
ΔGLS (%)	0.066	0.661		

*IABP* intra-aortic balloon pump, *ECMO* extracorporeal membrane oxygenation, *IVS* interventricular septum, *LVEDD* end-diastolic dimensions, *LA* left atrium, *EF* ejection fraction, *E* peak early diastolic mitral flow velocity, *A* peak late diastolic mitral flow velocity, *E′* Spectral pulsed-wave Doppler-derived early diastolic velocity from the septal mitral annulus, *GLS* global longitudinal strain.

ROC analysis was then performed and demonstrated that GLS at discharge with a cut-off value of 13.2% could effectively detect the GLS normalization at two years (sensitivity 82.2% and specificity 58.8%, AUC 0.758). In comparison, LVEF at discharge with a cut-off value of 54.5% could detect GLS normalization with inadequate power (sensitivity 72.4% and specificity 35.3%, AUC 0.616) (Fig. 3D).

## Discussion

Conflict results were reported in the previous case series of FM in terms of prognosis and LV functional recovery in the long-term^1,4,10,17–19^. Our FM patients received the treatments of Chinese protocol to in acute phase and led to favourable clinical outcomes in the term of in-hospital mortality^8,9^. The present study also found that this cohort of FM patients have much better long-term prognosis (all patients survived) than previous reports. During 2-year follow-up, both LVEF and LV chamber dimension of the patients are not significantly changed. The GLS has mild but significantly improved, and further found that GLS > 13.2% at discharge could predict normalization of GLS (≥ 17%) at two years (AUC, 0.758).

The present study included viral-FM adults who presented with acute onset of cardiac symptoms within 2 weeks and with distinct viral prodrome, excluding those with clinically suspected other forms of myocarditis than viral myocarditis. One of the aims is to exclude the potential cofounding effects from children and other forms of myocarditis. The other aim is to make the present study population comparable with the two previous studies, the 2000 McCarthy^19^ and the 2017 Ammariti study^4^, which were two important studies on prognosis of FM to date but with conflict results.

In terms of management, the Chinese protocol LSBCTR was used in these patients which includes timely circulatory support and immunomodulation therapy with sufficient doses of both glucocorticoid and immunoglobulin. Chinese treatment consists of two key approaches, MCS and immunomodulation therapy including steroids and intravenous immunoglobulins (IVIG). Temporary MCS has been widely accepted as an effective therapy in contemporary treatment of FM^7,17,20^. But our regimen indicated IABP is priority and if IABP is not enough to support sufficient circulation, ECMO will be added. Because the use of ECMO single will increase afterload which may cause failure of treatment, while IABP lowered afterload and it can be used alone.

This point of view was further supported by an newly published article in The New England Journal of Medicine^21^. Similarly, biventricular unloading, adequate systemic and coronary perfusion, and venous decongestion were emphasized as primary treatment goals. Thus, the importance of temporary devices such as IABP and intravenous ECMO in the treatment was highlighted. This novel comprehensive treatment regimen ensures high survival rate with excellent recovery of left ventricle function in acute phase. And its effects extends to subsequent follow-up duration under small dose of prednisone (20–30 mg/day) for 3 months and beta receptor blocker and ACE inhibitor.

For both FM patients and corresponding medical staffs, in-hospital mortality and long-term prognosis are two most important clinical parameters. 2 patients died during the hospitalization period before EMB and/or CMR were taken in the study. When these 2 patients were considered, the in-hospital mortality would be 4.2%, similar as that in previous study^8^. No death or heart transplantation occurred among these 46 survivors at 2-year, but longer follow-up is necessary to ascertain this favourable long-term survival.

The study by McCarthy et al. in 2000 included 15 patients with FM^19^. Among them, no death or transplantation occurred during an average follow-up of 5.6 years, which has led to the belief that FM was characterized by critical illness at presentation and higher in-hospital mortality will have excellent long-term survival. After that, 2 contemporary case series of FM published in 2017 and 2019, by Ammirati et al.^4,10^. Findings from the 2017 study supported the initial belief when only survival was considered, where all 4 deaths (11.8%) occurred during hospitalization with no cardiac deaths after acute phase^4^. However, the 2019 study challenged this belief. This study was the largest case series of FM reported to date with 108 FM adults, where cardiac death and heart transplantation rate was 19.5%, 31.5% and 41.4% at 60 days, 1 year and 7 years^10^. Our findings from the present study, although with only 2-year follow up data, are in line with the McCarthy and 2017 Ammirati study, and support the belief of better long-term survival of FM.

Previous studies, had revealed that FM survivors usually had steep improvement of LV function during hospitalization^11,22^. In the present study, although further improvement of LVEF in the whole population at 2-year was not observed, modest improvement of GLS was present. There were also no significant changes in LVEDD. However, a proportion of patients had LV dysfunction at 2-year, that was 22% assessed by LVEF (< 55%) and 37% by GLS (< 17%). Therefore, we confirmed that a relatively large proportion of patients with viral FM had residual LV dysfunction in the long-term. The outcomes and future functional changes of them should be further studied.

Our results were in line with the 2017 Ammirati study but at odds with the other two earlier studies^4,17,18^. In Ammirati study, a modest improvement of LVEF was observed, but the LVEF in 23% of patients remained at < 55% at a median follow-up of 22 months^4^. Another study by Ammirati in 2018 also reported 18.1% of patients with LVEF < 55% at about 200 days follow-up in a population called complicated AM, whose characteristics were close to FM^1^. In comparison, the earlier two studies reported substantial improvement or almost complete recovery of LV function in all survivors with FM^17,18^. However, these two studies had some discrepancies in the study population from the present and the 2017 Ammirati study, and included lower number of FM cases (≤ 14) and thus had inadequate statistic power^17,18^.

The present study also sought to ascertain whether early echocardiographic parameters can predict outcomes or LV functional recovery after discharge. Studies investigating the early prognostic predictors in FM are lacking. A study performed in children with myocarditis revealed that the presence of LVEF < 30% at admission was the major predictor for poor outcomes^23^. Another study found that the severity of AM at presentation was associated with outcomes within 3-months in adult survivors from AM^24^. In the international registry by Ammirati in 2019, LVEF at admission, dichotomized as ≤ 30%, did not correlate with both short-term and long-term outcomes in FM^10^.

The previous studies demonstrated that strains by 2-D STE was more sensitive in detecting cardiac dysfunction^13^ and could provide more information in myocarditis^11,14^. Therefore, in the present study, GLS was used to evaluate LV function other than LVEF. We found that both LVEF or GLS at admission did not correlate with LV function at two years detected by GLS, which is partly in accordance with the 2019 Ammirati study^10^. In comparison, there was an association between GLS at discharge and at two years. The patients with GLS < 13.2% at discharge was not likely to have GLS normalization at 2-year. We concluded that the severity of LV dysfunction at discharge but not that at presentation could predict LV functional recovery in the long-term and GLS could provide additional prognostic information than LVEF.

## Limitations

Firstly, we did not perform EMB in all patients. Therefore, we used the term viral myocarditis and data obtained from this study population may not be able to apply to lymphocytic myocarditis completely. However, it must be noted that EMB can’t be completed in many centres in the real world. The percentage of EMB in many reports of FM was also low^5,25^. Moreover, due to the low percentage of EMB and no molecular analysis for virus in the present study, the association between prognosis and the presence of virus could not be assessed. Despite these defects, the overall survival and functional recovery was benign in our cohort of patients. Thus, we challenged the necessity for timely EMB in myocarditis with fulminant presentation, as suggested by current consensus statement^26^, particularly if immunomodulation therapy could be standardized in treating viral FM as we did in the present study. Next, this is a retrospective study in only FM and no data from NFM are available to make comparison. Finally, due to ethical reasons, all patients in the present study received immunomodulation therapy, thus its efficacy for viral FM could not be adequately proved.

## Conclusions

Our data suggest that FM adults who have survived the acute episode treated with the Chinese protocol will have benign prognosis at 2-year when only survival is considered. LV function of them improved modestly as well at two years. However, LV function in a great proportion of them remains low at 2-year, particularly from the viewpoint of GLS. And patients with low GLS at discharge, implying those with poor recovery of LV function after acute episode, are more likely to have persistent LV dysfunction in the long-term. Whether subsequent heart transplantation might be necessary or even death might occur for FM patients treated with the Chinese protocols requires further study.

## Methods

### Study population, design and diagnosis of FM

Consecutive patients with surveyed acute myocarditis (the aged > 14 years) admitted to Tongji hospital (that is the tertiary referral hospital) in Hubei province China from March 2015 to December 2019 was retroactively screened. AM was defined by meeting one of the following criteria, (1) histologically proven myocarditis by EMB (Supplemental Fig. S1) or (2) elevated troponin level plus two “Lake Louise” CMR criteria being positive^27^. FM was then diagnosed if the following criteria were met: (1) the presence of viral prodromal signs/symptoms followed by acute onset of severe heart failure (HF) within less than two weeks; (2) severe hemodynamic compromise requiring inotropic agents, and even MCS supports^12,28^. In the setting of FM, CMR was usually performed before discharge. Moreover, the primary analysis of the study focused on viral myocarditis that was patients with distinct viral infection-like prodromal symptoms. And patients with clinical findings suggestive of a different pathogenesis as eosinophilic myocarditis were excluded.

**Figure 4:**
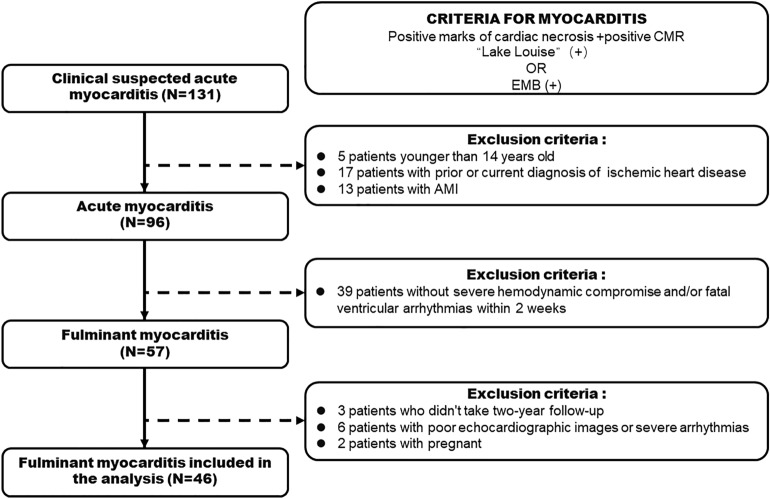
Flow diagram describing the selection protocol of 46 adult patients with FM from the 131 population with suspected AM in the present study.

According to the enrolling protocol described above (Fig. 4), 96 patients with AM were included and 55 of them were diagnosed as vial-FM. Among them, 3 patients who had no 2-year follow-up data, 6 patients with poor echocardiographic images or the presence of arrhythmias that interfered with strain analysis. Finally, the study population consisted of 46 patients with viral FM.

This research was approved by the ethics committee of Tongji Hospital, Tongji Medical College, Huazhong University of Science and Technology (TJ‑C20160202), and informed consent for this study was obtained from all participants. Moreover, the study was performed in accordance with the Declaration of Helsinki.

### Patient management

All patients in the study received the Chinese protocol, that is LSBCTR^12,29^. LSBCTR consists of (i) mechanical life support (positive pressure respiration, IABP with or without ECMO), (ii) immunomodulation therapy using sufficient doses of glucocorticoids and immunoglobulins, and (iii) application of neuraminidase inhibitors. The core concept of the protocol is to modulate the immune response and provide circulatory support to the deteriorated hemodynamic status. With the protocol, in-hospital mortality of FM was reduced from about 50% to < 5% and the hospitalization period was shortened to < 2 weeks^8,9^.

Temporary MCS, including the use of IABP and/or ECMO, is one of the core approaches in the protocol that provides important circulatory supports to the patients. MCS has been widely accepted in the modern management of FM and has greatly improved the FM outcomes since its introduction. In LSBCTR, IABP is usually the first line choice of MCS, and ECMO is added in combination with IABP in the setting of persistent heart failure and low cardiac output syndrome. Devices as Impella and other ventricular assistance devices were also reported to be used in FM but not routinely recommended in the LSBCTR due to controversial evidence^30,31^.

In comparison, immunomodulation therapy in the protocol was largely debated, and the application of neuraminidase inhibitors in FM was also not adequately evidenced. “The Myocarditis Treatment Trial” failed to support the use of glucocorticoids and IVIG as a routine treatment of FM but proved the efficacy of cytotoxic drugs that was otherwise not recommended in the LSBCTR. Moreover, the protocol suggests the use of neuraminidase inhibitors such as oral oseltamivir to reduce the cardiac damage caused by neuraminidase release^12,32^.

### Echocardiographic examination and strain analysis

All scans were performed by means of a 3.5 MHz transducer on a single commercially echocardiographic system (Vivid E9; GE Vingmed; Horten, Norway). The records of echocardiographic clips at admission, before discharge and at 2-year were obtained and analysed by a cardiologist experienced in echocardiographic imaging analysis. The last echocardiogram before discharge in FM patients was assessed GLS at discharge.

LVEF was calculated by the modified biplane Simpson’s method from apical 4- and 2-chamber views. An LVEF < 55% was considered incomplete recovery, and an LVEF < 40% was considered poor recovery of LV function. Other standard measurements were obtained according to the current guidelines of the American society of echocardiography/European association of cardiovascular imaging^15^.

GLSs were obtained by 2-D STE. Three consecutive cardiac cycles of 2-D images from apical 4-, 3-and 2-chamber views with optimized focus on LV were saved in digital format. Data processing was conducted off-line by using Echo Pac (version:113, 2017; GE Vingmed, Horten, Norway). Strain was quantified by automated function imaging analysis as previously described^11^ and was expressed as an absolute value.

### Statistical analysis

Continuous variables with a non-normal distribution were expressed as the median (interquartile ratio) or mean ± standard deviation, and categorical variables as percentages. The parameters of the two subgroups were compared by using paired-samples t test or Wilcoxon test as appropriate. Proportional differences were evaluated with X^2^-test or Fisher exact test. Relationships between two variables were analysed by means of linear regression and were expressed as Pearson correlation coefficients. Covariate was selected by clinical perspective and applied in the multivariate linear regression model. The associations between parameters during hospitalization or before discharge and GLS at 2-year follow-up were tested by univariate and multivariate linear regression analysis. The predictive value of the significant variables, as well as their diagnostic accuracy was assessed with receiver-operating characteristics (ROC) analysis.

All statistical tests were two-sided and a p value < 0.05 was considered to indicate statistical significance. Statistical analysis was performed using the SPSS Version 20.0 software package (SPSS 20.0, Chicago, IL, USA).

## Supplementary Information

Supplementary Information.

## Data Availability

The original contributions presented in the study are included in the article. Further inquiries can be directed to the corresponding author by wanghong2022gls@126.com.
