# Author Correction: Left ventricular function changes and echocardiographic predictors in adult survivors of fulminant myocarditis treated with the Chinese protocol

**DOI:** 10.1038/s41598-023-35114-7

**Published:** 2023-06-12

**Authors:** Luying Jiang, Kaiyue Zhang, Chunran Zhang, Yujian Liu, Jiangang Jiang, Daowen Wang, Houjuan Zuo, Hong Wang

**Affiliations:** 1 Division of Cardiology, Department of Internal Medicine, Tongji Hospital, Tongji Medical College, Huazhong University of Science and Technology, Wuhan, 430030 People’s Republic of China; 2 Hubei Key Laboratory of Genetics and Molecular Mechanisms of Cardiologic Disorders, Wuhan, 430030 Hubei Province People’s Republic of China; 3 The 3rd Department of Cardiology, The First Affiliated Hospital of The Medical College, Shihezi University, Shihezi, 832008 Xinjiang People’s Republic of China

Correction to: *Scientific Reports* https://doi.org/10.1038/s41598-023-33285-x, published online 18 April 2023

The original version of this Article contained an error in the spelling of the author Daowen Wang, which was incorrectly given as Dao Wen Wan.

The original Article has been corrected.

