# Supplementary material for: Left ventricular function changes and echocardiographic predictors in adult survivors of fulminant myocarditis treated with the Chinese protocol
Source: Sci Rep. 2023 Apr 18;13:6274. doi: 10.1038/s41598-023-33285-x (PMC10113373; doi:10.1038/s41598-023-33285-x)
Supplement: Supplementary file 1 — Supplementary Information. [file 41598_2023_33285_MOESM1_ESM.pdf]

Supplemental data

Supplemental Figure 1. Histological images of a patient with FM

(A and B) Hematoxylin and eosin (H&E) section (×40 magnification) of two endomyocardial biopsy (EMB) with FM. There is evidence of diffuse lymphoid infiltrates and neutrophilic infiltrates with edema and multifocal cardiomyocyte necrosis.

Supplemental Figure 2. Representative bull's-eye displays of GLSs in a 33-year woman (A and B) and A 29-year woman (C and D) with FM, respectively, upon discharge and two-year follow-up. A, significant reduction of PSLSs in basal-, mid-inferior, posterior and antero-septal segments, with the average GLS being 10.3%; B, complete normalization of GLS; C, significant reduction of PSLSs in basal-, mid-, apical-inferior, posterior and antero-septal segments at discharge, with the average GLS being 9.1%; D, the GLS remained at 9.7 %.

Supplemental Figure 3: Changes in LVEF during two-year follow-up in patients with FM. The proportion of FM patients with LVEF<40% at two-year follow-up was higher than discharge.

Supplemental Figure 1

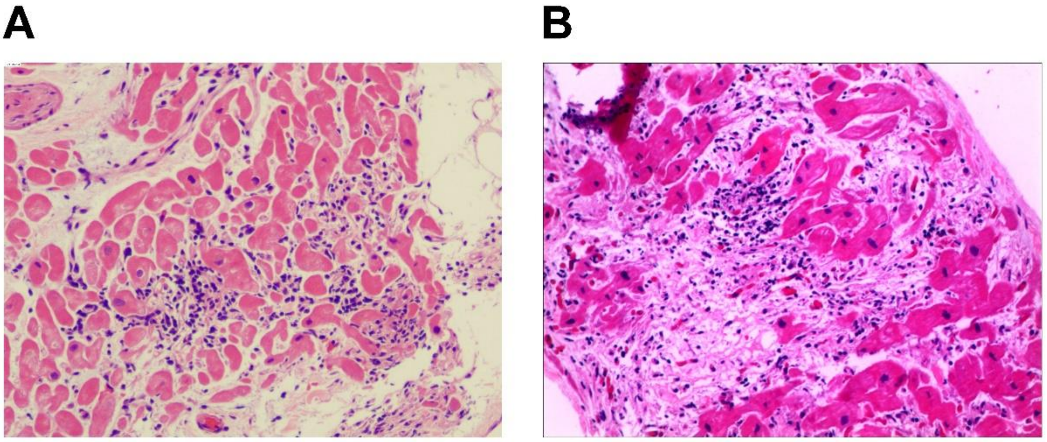

Supplemental Figure 2

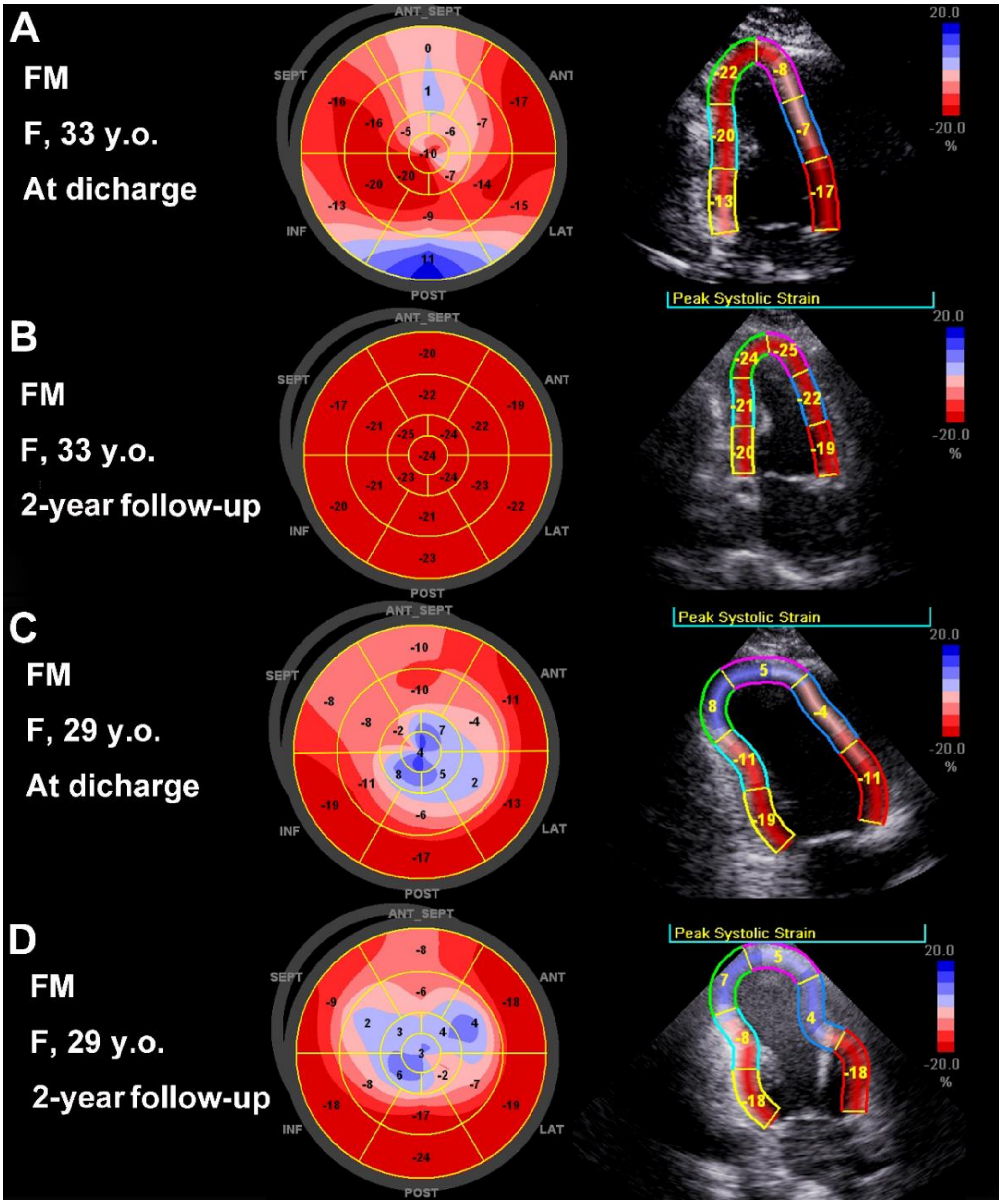

Supplemental Figure 3

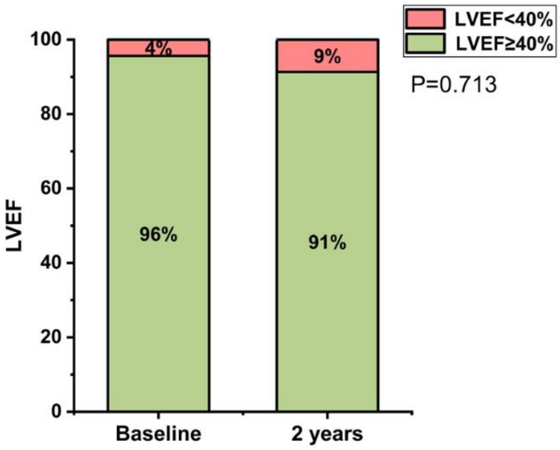

Supplemental Table 1 Clinical Presentations of 46 Patients with Fulminant Myocarditis

Clinical data	Value
Male-n (%)	23(50)
Female-n (%)	23(50)
Age (year)	33±13
Height (cm)	167.72±7.58
Weight (kg)	64.38±12.58
Body surface area (m^2)	1.69±0.19
Body mass index (kg/m^2)	22.78±3.47
Systolic blood pressure (mmHg)	112.54±11.27
Diastolic blood pressure (mmHg)	68.63±10.20
Heart rate (bpm)	74±11
**Biochemistry and Urinary Examination**	
CRP (mg/L)	11.35(3.58,39.25)
Peak troponin-T (pg/mL)	39649.75(14830.75,50000.00)
Troponin-T(pg/mL)	153.15(73.98，374.18)
NT-proBNP (pg/ml)	667.00(355.30,1468.25)
ALT (U/L)	59.00(33.75,106.50)
AST (U/L)	43.00(25.25,80.00)
Creatinine (μmol/L)	71.76±35.83
Lactic acid (mmol/L)	2.00±1.03
Glucose (mmol/L)	8.29±2.57
**Clinical presentation**	
Chest distress-n (%)	34(74)
Chest painc-n (%)	15(33)
Fever-n (%)	21(46)
Vomit-n (%)	17(37)
Diarrhea-n (%)	9(20)
Associated autoimmune disorders-n (%)	11(24)
Arrhythmology-n (%)	38(83)
**ECG**	
T-wave inversion-n (%)	16(35)
ST segment elevation -n (%)	18(39)
Other abnormal ST-T segment-n (%)	20(43)
Ventricular arrhythmia -n (%)	19(41)
Bundle branch block -n (%)	12(26)

III atrioventricular block-n (%)	8(17)
**Treatment**	
IABP -n (%)	46(100)
Days of IABP use(day)	5.95±2.17
ECMO -n (%)	9(20)
Days of ECMO use(day)	4.78±1.30
CVVH -n (%)	23(50)
Days of CVVH use(day)	1.57±0.59
Glucocorticoid(mg)	975.61±468.59
Days of glucocorticoid use(day)	8.00±3.13
R-globulin(g)	57.80±29.01
Days of r-globulin use(day)	6.34±2.88
**Post-discharge medication**	
Glucocorticoid -n (%)	20(43)
ACEI/ARB -n (%)	27(59)
Coenzyme Q10 -n (%)	45(98)
Trimetazidine -n (%)	44(96)
β-blocker -n (%)	32(70)

CRP, C-reactive protein; ALT,alanine aminotransferase; AST,aspartate aminotransferase; IABP, intra-aortic balloon pump; ECMO, extracorporeal membrane oxygenation;CVVH, continuous venovenous hemofiltration; ACEI/ARB, angiotensin-converting enzyme inhibitor or angiotensin receptor blocker

Supplemental Table 2 Comparison of global and segmental PSLSs between at discharge and at two-year follow-up

	At discharge	At two-year follow-up	P Value
GLS PSLS (%)	15.40±3.89	17.24±2.89	0.002
Mid PSLS (%)	15.57±4.40	17.28±3.48	0.039
Basal PSLS (%)	13.56±4.34	15.40±3.20	0.023
Apical PSLS (%)	18.19±6.92	18.69±5.58	0.51
Ant Sept(%)	14.50±5.95	17.40±3.97	0.029
Ant (%)	14.72±2.22	15.76±4.51	0.431
Lat(%)	12.88±6.57	15.22±6.59	0.105
Post(%)	12.19±9.38	15.85±5.77	0.014
Inf(%)	17.48±4.55	18.11±3.60	0.69
Sept(%)	15.64±3.83	15.69±4.45	0.755

GLS, global longitudinal strain; PSLS, peak systolic longitudinal strain; Global PSLS, including apical, mid and basal PSLS%

Supplemental Table 3 Clinical Presentations of FM patients at admission and at discharge

Clinical data	At admission	At discharge	P
Male-n (%)	23(50)	N/A	N/A
Female-n (%)	23(50)	N/A	N/A
Age (year)	33±13	N/A	N/A
Height (cm)	167.72±7.58	N/A	N/A
Weight (kg)	64.38±12.58	N/A	N/A
Body surface area (m^2)	1.69±0.19	N/A	N/A
Body mass index (kg/m^2)	22.78±3.47	N/A	N/A
Systolic blood pressure (mmHg)	91.54±13.26	112.54±11.27	0.001
Diastolic blood pressure (mmHg)	59.26±7.73	68.63±10.20	0.035
Heart rate (bpm)	102.35±15.62	74.00±11.00	0.025
Biochemistry Examination			
CRP (mg/L)	38.75(7.65,102.48)	11.35(3.58,39.25)	<0.001
Troponin-T (pg/mL)	33981.95(13588.00,50000.00)	153.15(73.98,374.18)	<0.001
NT-proBNP (pg/ml)	13643.00(4839.75,31927.50)	667.00(355.30,1468.25)	<0.001
ALT (U/L)	147.00(69.75,318.00)	59.00(33.75,106.50)	<0.001
AST (U/L)	330.00(171.00,563.25)	43.00(25.25,80.00)	<0.001
Creatinine (μmol/L)	82.72±29.44	71.76±35.83	0.345
Lactic acid (mmol/L)	2.00±1.03	N/A	N/A

ALT,alanine transaminase; AST,aspartate aminotransferase; NT-proBNP=N-terminal pro-B-type natriuretic peptide

Supplemental Table 4 Comparison of echocardiographic parameters at admission and at discharge

Parameters	At admission	At discharge	P
IVS systolic (cm)	1.08±0.19	1.25±0.21	0.241
LVEDD (cm)	4.61±0.54	4.78±0.55	0.352
LA diameter (cm)	3.03±0.37	3.27±0.57	0.163
EF (%)	32±13	57.20±8.29	<0.001
GLS PSLS (%)	8.32±2.78	16.19±4.00	<0.001
Mid PSLS (%)	7.74±5.26	15.57±4.40	<0.001
Basal PSLS (%)	7.31±2.62	13.56±4.34	<0.001
Apical PSLS (%)	8.67±3.51	18.19±6.92	<0.001

IVS, interventricular septum; LVEDD, LV end-diastolic dimensions; LA, left atrium; EF, ejection fraction; PSLS, peak systolic longitudinal strain; GLS, global peak systolic longitudinal strain.

Supplemental Table 5 Clinical and echocardiographic parameters of the 4 patients with poor recovery of LV function

Number	Age /Sex	Recent history	ECG change	Troponin (Peak/discharge)	NT-proBNP	LVEF (discharge/ follow-up)	GLS (discharge /follow-up)	ECMO	IABP	Glucocorticoid	r-globulin
1	26 /F	Chest distress, Vomit	ST elev, VT	50000/ 718.2 (pg/mL)	949 (pg/mL)	38/34 (%)	16.7/ 12.2 (%)	0	9(day)	1440 mg/ 12day	85g/ 9day
2	50 /M	Fever	ST elev, AVB, VT	50000/ 50.7 (pg/mL)	1472 (pg/mL)	49/36 (%)	15.7/ 14.6 (%)	0	8(day)	1280 mg/ 14day	105g/ 13day
3	29 /F	Chest painc， Chest distress	T inv	50000/ 532 (pg/mL)	2259 (pg/mL)	32/37 (%)	9.1/ 9.7 (%)	0	11 (day)	2840 mg/ 18day	115g/ 13day
4	57 /M	Chest painc, Chest distress, Vomit	T inv	29234.9/ 4316.7 (pg/mL)	2993 (pg/mL)	55/26 (%)	10/ 11.2 (%)	4 (day)	5(day)	780 mg/ 4day	30g/ 3day

ST elev, ST elevation; T inv., T wave inversion;AVB, atrioventricular block; VT, ventricular tachycardia; IABP, Intra-aortic balloon pump; ECMO, Extracorporeal Membrane Oxygenation; NT-proBNP, N-terminal prohormon B-type natriuretic peptide.

Supplemental Table 6 EMB and CMR found of the 4 patients with poor recovery of LV function

Number	Age /Sex	LVEF (%)	LGE mass (g)	LGE mass %	T1 mapping /max	ECV /max	T2 mapping /max	Inflammatory infiltrate*	Extent of necrosis *	Dallas criteria	CD3/ CD68 infiltrate
1	26 /F	38.1	47.5	58.4	1457 / 1565	0.33 / 0.39	52 / 57	3	2	Active M CD3>14 mm^2, CD68>14 mm^2	CD68> CD3
2	50 /M	28.9	32.5	42.8	1618 / 2045	0.45 / 0.67	50 / 67	2	3	Active M CD68>14 mm2	CD68> CD3
3	29 /F	34.7	58.7	53.9	1586 / 1743	0.50 / 0.66	62 / 76	3	1	Active M CD3>14 mm^2, CD68>14 mm^2	CD3> CD68
4	57 /M	40.2	62.6	64.97	1547 / 1664	0.41 / 0.47	57 / 63	NA	NA	NA	NA

*0= none, 1= mild, 2= moderate, 3= severe

M, myocarditis NA, not available

LVEF, left ventricular ejection fraction; LGE, late gadolinium enhancement; ECV, extracellular volume
